# Worsening Disability and Hospitalization Risk in Sarcopenic Obese and Dynapenic Abdominal Obese: A 5.5 Years Follow-Up Study in Elderly Men and Women

**DOI:** 10.3389/fendo.2020.00314

**Published:** 2020-06-30

**Authors:** Andrea P. Rossi, Silvia Urbani, Francesco Fantin, Nicole Nori, Piero Brandimarte, Angela Martini, Elena Zoico, Gloria Mazzali, Alessio Babbanini, Valentina Muollo, Mauro Zamboni

**Affiliations:** ^1^Division of Geriatric, Department of Medicine, University of Verona, Verona, Italy; ^2^Department of Medicine, Clinical and Experimental Biomedical Sciences, University of Verona, Verona, Italy

**Keywords:** sarcopenic obesity, muscle strength, dynapenia, disability, hospitalization

## Abstract

**Background/Objectives:** A general lack of studies comparing the effect of both dynapenic abdominal obesity and sarcopenic obesity on worsening disability and hospitalization risk should be recognized. The aim of the current study was to evaluate, with a 5.5-year follow-up, the prognostic value of sarcopenic obesity and dynapenic abdominal obesity definitions on worsening disability and hospitalization risk in a sample of older adults.

**Subjects/Methods:** In 177 women and 97 men aged 68–78 years, the following outcomes were evaluated at baseline: appendicular skeletal muscle mass (ASMM), percent fat mass (FM%), leg isometric strength, body mass index (BMI), lipid profile, vitamin D3, albumin, fibrinogen, glycemia, physical activity level, income, smoking status, and comorbidities. The rate of reported disabilities and hospitalization were also assessed at baseline, 1, 2, 3, and 5.5-years follow-up. The study population was classified into: (i) non-sarcopenic/obese (NS/O), sarcopenic/non-obese (S/NO), sarcopenic/obese (S/O), non-sarcopenic/non-obese (NS/NO, reference category) according to relative ASMM/FM% tertiles; (ii) non-dynapenic/abdominal obese (ND/AO), dynapenic/non-abdominal obese (D/NAO), dynapenic/abdominal obese (D/AO), non-dynapenic/non-abdominal obese (ND/NAO, reference category) according to muscle strength/waist circumference tertiles.

**Results:** The prevalence of D/AO and S/O was 12.0 and 8.0%, respectively. Only 2 subjects were both D/NAO and S/O (0.8%). D/NAO subjects showed a worsening disability risk of 1.69 times (95% CI: 1.11–2.57), ND/AO subjects showed a 2-fold increased risk (95% CI: 1.34–2.98), while being D/AO more than trebled the risk, even after adjustment for confounding factors (HR: 3.39, 95%; CI: 1.91–6.02). By dividing the study population according to the relative ASMM/FM% tertiles, no groups showed an increased risk of worsening disability. The hospitalization risk, even after adjustment for potential confounders, was 1.84 (95% CI: 1.06–3.19) for D/AO. Dividing the study population according to the relative ASMM/FM% tertiles, no groups showed increased risk of hospitalization.

**Conclusions:** Our results showed that dynapenic abdominal obesity and sarcopenic obesity seem to indicate two distinct phenotypes associated with different health risk profiles. The distribution of participants in waist circumference and muscle strength tertiles allowed for a more accurate risk stratification for worsening disability and hospitalization.

## Introduction

In the elderly population, the increase in the prevalence of obesity and concomitant age-related skeletal muscle loss leads to a condition called sarcopenic obesity (SO), which is characterized by an imbalance between muscle mass loss and fat mass accumulation (FM).

The term SO was first introduced by Baumgartner et al. (1) as concurrence of low muscle mass, expressed as appendicular skeletal muscle mass divided by height squared (ASMM/h^2^), and a high amount of FM (i.e., percentage of body fat >28% in men and 40% in women or high values of BMI). Several other definitions considering FM and skeletal muscle mass as determined by DXA have been proposed, determining a high degree of variability in SO prevalence, depending on current research definitions (2, 3). Moreover, waist circumference or waist to hip ratio were at times used instead of FM (4–6).

SO is strongly associated with physical disability in older adults (4–6).

Only a few studies have examined the combined effect of obesity and muscle mass decline on disability trajectories in the elderly population, and provide conflicting results (7, 8).

Over the years, other definitions of SO have been proposed, focusing on muscle strength impairment, rather than muscle mass, and the concept of dynapenic abdominal obesity (DAO) has been introduced (9–12). The dynapenic abdominal obese phenotype shows the worst profile, as it is exposed to a higher risk of developing mobility, disability and hospitalization (13, 14). However, prospective studies comparing the worsening disability and hospitalization risk in both SO and DAO are still lacking.

Therefore, the aim of the current study is to determine the prognostic value of both dynapenic abdominal obesity and sarcopenic obesity and risk of hospitalization and worsening disability in a sample of older adults.

## Materials and Methods

### Subjects

The study population was randomly selected from the lists of 11 general practitioners (GP) from Verona. Subjects were eligible if they had no physical cognitive impairment. Anthropometry and disability were evaluated at baseline in a cohort of community-dwelling elderly men and women. None of the subjects were involved in physical activity programs more than once a week during the study. Exclusion criteria included heart failure (NYHA ≥2), disabling knee osteoarthritis, cancer, renal failure and serious pulmonary disease. Subjects who had lost more than 5% of their body weight during the year before the study start were also excluded. The GP lists provided 3,000 potential subjects, while 1,145 were finally chosen. Six hundred and twenty five subjects were potentially eligible and 434 provided contact information, 28 were unable to be contacted, 81 refused and 51 were ineligible. A total of 274 participants, 177 women and 97 men, aged 66–78 years, were eligible and gave their informed consent, which represents 63.1% of the individuals who had initially provided contact information.

The study was approved by the Ethics Committee of the University of Verona.

### Anthropometric Measures

Body weight was measured to the nearest 0.1 kg, and to the nearest 0.5 cm using a stadiometer (Salus scale and stadiometer, Milan, Italy). BMI was derived from body weight divided by stature (kg/m^2^). Waist circumference (WC) was obtained with a measuring tape at the level of the narrowest part of the torso as viewed anteriorly.

### Muscle Strength

The maximal voluntary isometric strength of the dominant knee extensor flexed at 90 degrees was tested by a Spark Handheld Dynamometer model 160 (Spark Instruments & Academic Inc., Iowa City, IA, USA).

During 5 min of a warm-up phase, the subjects were familiarized with the trials at a submaximal level before starting the test. Thereafter, the subjects performed three maximal isometric contractions interspersed by 60 s of recovery. Strength was recorded in kg and the mean peak torque of three trials was calculated and used in the analyses, as reported elsewhere (13).

### Dual Energy X-Ray Absorptiometry (DXA)

Total FM and appendicular skeletal muscle mass (ASMM) were determined using DXA (Hologic QDR 2000 fan beam densitometer with software version 7.2) as described elsewhere in more detail (15, 16). In our lab the coefficient of variation in a sample of 11 subjects aged between 65 and 75 for fat mass and lean mass were 2 and 1.3%, respectively.

### Classification of Groups

#### Definition of Sarcopenic Obesity

Sex-specific tertiles of relative ASMM and FM% were created. Subjects were classified as sarcopenic if their relative ASMM was below the lowest tertile (7.00 kg/m^2^ for men and 5.18 kg/m^2^ for women) and as obese if their FM% was in the highest tertile (above 31.17% for men and 44.01% for women). Based on the combination of relative ASMM and FM% cut-off values, subjects were further classified into four groups: sarcopenic obese (S/O), sarcopenic non-obese (S/NO), obese (NS/O), and non-obese (NS/NO).

#### Definition of Dynapenic Visceral Obesity

Tertiles of muscle strength in men and women were created in order to obtain equal groups as much as possible, in accordance with previous studies (10, 12, 13). Subjects belonging to the lowest tertile of leg muscle strength (20.58 kg for men and 11.66 kg for women) were classified as dynapenic, while those in the second and third tertiles were classified as non-dynapenic. WC tertiles were used to divide subjects up as abdominal obese or non-abdominal obese (100.00 and 87.00 cm, respectively, in men and women).

Individuals were classified into four groups based on sex-specific strength and WC tertiles: dynapenic abdominal obese (D/AO), non-dynapenic abdominal obese (ND/AO), dynapenic non- abdominal obese (D/NAO), non-dynapenic non-abdominal obese (ND/NAO).

### Health Status

The presence of acute or chronic conditions, smoking status, diabetes, hypertension and hypercholesterolemia was determined during the anamnestic data collection. Subjects underwent a careful clinical investigation at the baseline assessment, and at the 1, 2, 3, and 5.5-y follow-up. Information regarding hospital admissions was obtained for each subject through their GPs' records. Hospitalization was defined as a stay of at least 48 h in an acute-care hospital. Chronic conditions assessed included: chronic obstructive pulmonary disease (COPD) and asthma, hypercholesterolemia, degenerative joint disease, cardiovascular disease, diabetes, and hypertension.

### Reported Disability

Reported disability was defined according to Langlois et al., based on the Activity of Daily Living scale (ADLs), the Rosow-Breslau physical function items and Instrumental Activity of Daily Living scale (IADLs), as described elsewhere in more detail (13, 17).

Any change in reported disability score between baseline and follow-up evaluation were assessed and the study population was then divided into two categories: unchanged and worsened, defined by the loss of one or more points over the follow-up period.

### Dietary Intake

A professional experienced dietician from the Department of Nutrition performed a 24-h dietary recall assessing the dietary habits of each participant. Daily intake of energy, fat, carbohydrate, and protein based on the tables furnished by the Italian National Institute of Nutrition was then calculated using a special software (18).

### Biochemical Measures

After overnight fasting, baseline blood samples were obtained from each subject.

Glucose was measured by using a compact chemistry analyzer method (Ektachem DT-60; Eastman Kodak, Inc., Rochester, NY) with interassay CV of 2% (19).

Triglycerides and cholesterol were determined using a compact chemistry analyzer (Eastman Kodak) method, and an inter-assay coefficient of variation of 2.2% for triglycerides and 2% for cholesterol was found. Dextran-magnesium precipitation was used for high-density lipoprotein separation (19). Calculated low density lipoprotein levels was derived using the Friedewald formula. Albumin was determined using a calorimetric test (Vitros 950 ALB slides; J&J Health, Cone Systems, Piscataway, NJ, USA); the color complex formed was measured by reflectance spectrophotometry. The sensitivity of the assay has been shown to be 10 gL^−1^; intra-assay CV were 1.3–1.5%. 25-hydroxyvitamin D (25[OH] D) level was measured as described elsewhere (20).

### Statistical Analysis

Results are shown as mean and SD. Preliminary Kolmogorov-Smirnov test for normality was performed on anthropometric variables (separately in males and females). None of the tests were significant, so repeated measures of analysis of variance (ANOVA) were performed.

In order to assess group differences at baseline, the study population was divided up based on muscle strength/WC and ASMM/FM% measurement, and a one-way ANOVA was performed. Chi-square tests were used for categorical variables.

One way-ANOVA was used to test group differences at baseline between subjects with or without hospitalizations and subjects with stable/worsened disability.

Cox regression was performed to assess worsening disability risk and hospitalization risk, at each follow-up period, dividing the population up into groups based on muscle strength/WC and ASMM/FM% tertiles. Both unadjusted and adjusted Hazard ratios were calculated for gender and age. Further adjustment for presence of disability at baseline, income, alcohol and smoking habit, fibrinogen, vitamin D3 level, physical activity level and comorbidity (COPD, stroke diabetes, hypercholesterolemia, and cardiovascular disease) was also performed (Model 3).

A significance level of 0.05 was used throughout the study. All statistical analyses were performed using SPSS (version 21.0 for Windows) (21).

## Results

Tables 1, 2 show the main baseline characteristics of the study population (mean ± SD), according to muscle strength/WC tertiles groups and ASMM/FM% tertiles groups, respectively.

**Table 1:** Baseline characteristics of the study population according to muscle strength/WC.

	**Non-dynapenic/non-abdominal obese (*n* = 123) x ± SD (min–max)**	**Dynapenic/non-abdominal obese (*n* = 57) x ± SD (min–max)^a^**	**Non-dynapenic/abdominal obese (*n* = 61) x ± SD (min–max)^a^**	**Dynapenic/abdominal obese (*n* = 33) x ± SD (min–max)^a^**	***P***
Age (years)	71.69 ± 2.29 (67–78)	72.23 ± 2.04 (69–78)	71.00 ± 2.30 (66–76)	72.09 ± 2.41 (68–76)	<0.05
Sex (female) *n* (%)	77 (62.6%)	39 (68.4%)	41 (67.2%)	20 (60.6%)	NS
Height (m)	1.61 ± 0.09 (1.42–1.90)	1.60 ± 0.08 (1.40–1.85)^b^	1.63 ± 0.10 (1.41–1.86)^b^	1.60 ± 0.08 (1.41–1.78)^b^	NS
Weight (kg)	65.21 ± 9.37 (43.30–86.50)	62.46 ± 10.04 (45.40–89.30)^b^	83.56 ± 13.33 (63.50–118.90)^d^	75.18 ± 8.1 (56–95.20)^d^	<0.001
BMI (kg/m^2^)	25.11 ± 2.72 (18.74–31.40)	24.21 ± 2.75 (18.19–30.19)^b^	31.60 ± 4.22 (25.47–50.58)^d^	29.23 ± 2.86 (23.17–35.61)^d^	<0.001
Waist circumference (cm)	83.57 ± 8.38 (64.50–100)	80.64 ± 8.58 (66–98)^b^	97.50 ± 9.43 (87–124)^d^	97.50 ± 6.35 (87–110)^d^	<0.001
Total fat-free mass (kg)	40.38 ± 8.64 (27.23–61.28)	38.03 ± 8.97 (27.38–58.31)	45.44 ± 10.85 (29.32–68.06)^c^	43.48 ± 81.76 (31.66–62.37)	<0.001
Total fat mass (kg)	21.46 ± 5.57 (5.52–33.94)	21.30 ± 54.64 (11.59–32.89)	34.12 ± 8.15 (22.39–61.06)°	28.49 ± 5.25(19.77–38.14)°	<0.001
Fat mass (%)	33.89 ± 8.32 (8.4–48.2)	35.20 ± 8.22 (18.6–50.7)	42.05 ± 8.11 (26.2–56.5)^d^	38.75 ± 7.06 (27.2–49.7)^c^	<0.001
Appendicular muscle mass (kg)	15.90 ± 3.97 (8.98–25.09)	14.85 ± 4.26(9.95–24.68)	18.13 ± 4.99 (11.61–29.43)^c^	17.13 ± 3.85 (11.24–24.89)	<0.001
ASM/h^2^ (kg/m^2^)	6.05 ± 1.08 (3.98–9)	5.69 ± 1.16(3.99–8.48)^b^	6.75 ± 1.24 (5.03–9.81)^c^	6.62 ± 1.10 (4.54–8.82)^c^	<0.001
Glycemia (mg/dl)	98.07 ± 25.52 (73–281)	97.05 ± 15.63 (80–187)	105.55 ± 24.22 (82–205)^d^	105.19 ± 35.88 (70–254)^d^	NS
Total cholesterol (mg/dl)	239.08 ± 38.37 (147–375)	238.33 ± 37.72 (161–322)	235.09 ± 34.23 (164–307)	244.63 ± 45.59 (148–326)	NS
HDL cholesterol (mg/dl)	62.97 ± 14.56 (22–111)	66.33 ± 16.95 (36–106)	56.52 ± 14.70 (34–95)^c^	56.18 ± 15.56 (31–98)^b^	=0.001
Triglycerides (mg/dl)	128.70 ± 53.22 (39–292)	118.78 ± 47.44 (49–264)	160.49 ± 73.70 (55–331)^c^	180.09 ± 88.41 (74–474)^d^	<0.001
Albumin (g/L)	43.76 ± 2.72 (36.6–52.3)	43.41 ± 3.42 (34–47.9)^d^	43.49 ± 3.78 (35.5–55)	43.37 ± 4.61 (23–48.1)^b^	NS
Vitamin D (ng/dl)	48.26 ± 28.51 (11–169)	42.67 ± 21.06 (1–95)^b^	46.40 ± 22.65 (11–108)	38.00 ± 44.03 (1–265)^b^	NS
Fibrinogen (mg/dl)	293.94 ± 63.32 (151–501)	310.32 ± 55.36 (208–480)	315.33 ± 66.83 (205–547)^b^	314.50 ± 49.58 (217–460)^b^	NS
ADL (1–4)	3.77 ± 0.44 (1–4)	3.45 ± 0.74 (1–4)^c^	3.43 ± 0.73 (1–4)^d^	3.19 ± 0.78 (1–4)^d^	<0.001
Right leg strength at dynamometer (kg)	15.33 ± 5.21 (8.33–30)	7.63 ± 3.16 (2.33–15)^d^	16.29 ± 6.27 (8.33–32)	8.33 ± 3.76 (3.66–15)^d^	<0.001
Daily energy intake (kcal/day)	1785.03 ± 614.69 (885–3019)	1810.88 ± 512.62 (885–3019)	1859.63 ± 507.16 (978–3077)	1702.58 ± 374.28 (893–2497)	NS
Daily carbohydrates intake (kcal/day)	219.84 ± 68.43 (56.1–392.3)	233.41 ± 69.13 (85.5–445.2)	222.92 ± 65.41 (104.2–434.2)	214.28 ± 57.18 (86.2–355.8)	NS
Daily protein intake (kcal/day)	63.90 ± 24.07 (26.6–178.6)	65.97 ± 21.99 (29.9–117.5)	62.28 ± 20.56 (31–125.4)	67.28 ± 20.57 (32.9–108.6)	NS
Daily fat intake (kcal/day)	56.79 ± 22.53 (18.3–149.7)	57.08 ± 21.99 (21.3–121.2)	63.17 ± 23.24 (22.2–149.7)^b^	56.28 ± 17.70 (23.9–86.3)	NS

*ASMM, appendicular skeletal muscle mass; ADL, Activity Daily Living; WC, Waist Circumference; BMI, Body Mass Index; ASMM/h^2^, appendicular skeletal muscle mass/height^2^*.

a *In comparison with reference category (non-dynapenic/non-abdominal obese)*.

b *P < 0.05*.

c *P < 0.01*.

d *P < 0.001*.

**Table 2:** Baseline characteristics of the study population according to ASMM/FM% groups.

	**Non-sarcopenic/non-obese (*n* = 116) x ± SD (min–max)**	**Sarcopenic/non-obese (*n* = 68) x ± SD (min–max)^a^**	**Non-sarcopenic/obese (*n* = 68) x ± SD (min–max)^a^**	**Sarcopenic/obese (*n* = 22) x ± SD (min–max)^a^**	***P***
Age(years)	71.61 ± 2.42 (66–78)	71.92 ± 1.66 (69–76)	71.75 ± 2.58 (67–78)	71.35 ± 2.35 (67–76)	NS
Sex (female) *n* (%)	73 (63.5)	44 (64.7)	44 (67.6)	14 (60.9)	NS
Height (m)	1.60 ± 0.09 (1.40–1.85)	1.62 ± 0.08 (1.42–1.90)	1.59 ± 0.09 (1.41–1.79)	1.62 ± 0.10 (1.47–1.86)	NS
Weight (kg)	68.34 ± 11.21 (43.3–99.5)	61.36 ± 9.46 (45.40–86.2)^d^	80.87 ± 13.04 (58.2–118.9)	70.77 ± 10.9 (56.2–98.7)	<0.001
BMI (kg/m^2^)	26.35 ± 3.02 (18.74–33.73)	23.01 ± 2.01 (18.19–27.6)^d^	31.68 ± 3.86 (26.78–50.58)^d^	26.55 ± 1.89 (23.34–30.58)	<0.001
Waist circumference (cm)	86.97 ± 9.54 (64.5–109)	80.05 ± 9.11 (66–102)^d^	97.82 ± 10.56 (77–124)^d^	88.76 ± 8.97 (75–110)	<0.001
Total fat-free mass (kg)	43.01 ± 9.42 (28.47–68.06)	38.22 ± 8.83 (27.23–61.28)^d^	42.83 ± 9.73 (29.16–65.3)^d^	38.45 ± 8.94 (28.86–55.17)^b^	<0.01
Total fat mass (kg)	22.0 ± 5.47 (8.58–36.35)	19.87 ± 4.78 (5.02–30.81)^c^	34.23 ± 7.61 (22.28–61.06)^d^	28.67 ± 4.13 (21.62–40.52)^d^	<0.001
Fat mass (%)	33.12 ± 7.47 (15.60–43.5)	33.6 ± 8.02 (8.4–43.6)	43.43 ± 7.14 (31.7–56.5)^d^	42.03 ± 6.13 (32–50.7)^d^	<0.001
ASMM (kg)	17.28 ± 4.38 (10.44–29.43)	14.58 ± 3.94 (8.98–23.02)^d^	17.05 ± 4.41 (11.11–26.71)	14.6 ± 3.88 (10.49–22.63)^c^	<0.001
ASMM/h^2^ (kg/m^2^)	6.59 ± 1.13 (4.58–9.81)	5.41 ± 0.94 (3.98–7.02)^d^	6.6 ± 1.1 (5.24–9.03)	5.42 ± 0.91 (4.28–6.89)^d^	<0.001
Glycemia (mg/dl)	102.17 ± 31.08 (70–281)	96.04 ± 17.92 (73–206)	102.33 ± 20.16 (80–205)	95.95 ± 16.28 (9–161)	NS
Total cholesterol (mg/dl)	238.43 ± 34.88 (147–321)	239.52 ± 37.25 (155–326)	241.27 ± 37.88 (148–313)	230.13 ± 52.13 (161–375)	NS
HDL cholesterol (mg/dl)	61.07 ± 17.02 (22–111)	66.13 ± 15.47 (31–106)^b^	58.38 ± 12.9 (35–99)	58.47 ± 15.06 (36–93)	<0.05
Triglycerides (mg/dl)	144.15 ± 64.37 (39–319)	123.44 ± 58.83 (49–344)^b^	147 ± 72.47 (55–474)	144.87 ± 58.8 (52–285)	NS
Albumin (g/L)	43.93 ± 3.26 (36.6–55)	43.98 ± 2.74 (38.5–52.3)	42.48 ± 2.74 (35.5–50.1)	43.85 ± 1.94 (40.6–47.8)	<0.01
Vitamin D (ng/dl)	44.56 ± 27.46 (1–137)	45.07 ± 40.89 (12–165)	38.86 ± 24.73 (1–115)	39.43 ± 13.75 (16–70)	<0.05
ADL (1–4)	1.37 ± 0.68 (1–4)	1.37 ± 0.57 (1–3)	1.62 ± 0.69 (1–4)^b^	1.61 ± 0.72 (1–3)	<0.05
Right leg strength at dynamometer (kg)	13.77 ± 6.74 (3–31.5)	12.24 ± 5.83 (2.33–29)	13.11 ± 5.51 (4–32)	12.21 ± 5.6 (4–22)	NS
Daily energy intake (kcal/day)	1703.68 ± 528.35 (709–3151)	1829.21 ± 487.18 (978–3019)	1873.45 ± 542.73 (912–3829)^b^	1806.86 ± 461.90 (1003–2595)	NS
Daily carbohydrates intake (kcal/day)	207.84 ± 66.03 (56.1–358.0)	229.71 ± 74.20 (93.9–445.2)	229.96 ± 62.10 (104.2–392.3)^b^	219.91 ± 52.83 (126.1–313.0)	NS
Daily protein intake (kcal/day)	64.07 ± 23.95 (26.6–178.6)	68.02 ± 21.18 (33.4–117.4)	65.89 ± 19.95 (31–117.5)	62.70 ± 23.17 (29.9–125.4)	NS
Daily fat intake (kcal/day)	56.79 ± 22.53 (18.3–118.2)	57.08 ± 21.99 (29.8–134.9)	63.17 ± 23.24 (21.3–149.7)	56.28 ± 17.70 (21.3–149.7)	NS

*ASMM, appendicular skeletal muscle mass; FM%, percentual fat mass; ADL, Activity Daily Living; ASMM/h^2^, appendicular skeletal muscle mass/height^2^; BMI, Body Mass Index*.

a *In comparison with reference category (Non-sarcopenic/non-obese)*.

b *P < 0.05*.

c *P < 0.01*.

d *P < 0.001*.

At baseline, D/AO, D/NAO, and ND/AO subjects showed higher disability compared to ND/NAO. D/AO and ND/AO showed higher BMI, WC, FM%, glycemia, triglycerides, fibrinogen and lower HDL compared to ND/NAO. ND/AO showed higher daily fat intake compared to ND/NAO. Moreover, D/AO and D/NAO showed lower albumin and vitamin D levels compared to other groups.

S/O showed higher BMI and FM% and lower ASMM compared to NS/NO. S/NO showed lower BMI, WC, total fat mass, ASMM, ASMM/h^2^, triglycerides, and higher HDL cholesterol compared to ND/NO (Table 2). NS/O showed higher daily energy and carbohydrates intake compared to NS/NO.

Prevalence of D/AO and S/O was 12.0 and 8.0%, respectively. Only 2 subjects out of 274 were both D/NAO and S/O (0.7%).

One hundred and eleven subjects (40.5%) showed worsening disability, with a decline in one or more items of the reported disability score over the 5.5-y follow-up. Subjects experiencing worsening disability presented higher BMI at baseline (data not presented in the Table).

Classifying the study population based on WC and strength in a Cox regression analysis, with ND/NAO as the reference category, the risk of worsening disability, after adjustment for gender and age, was 1.84 (95% CI: 1.25–2.72) for the D/NAO group; ND/AO subjects showed a 1.96 increased risk (95% CI: 1.35–2.86) and D/AO a 3.61 increased risk (95% CI: 2.12–6.15). After adjusting for other potential confounders, HR was 2.00 (95% CI: 1.34–2.98) for the ND/AO group and 3.39 (95% CI: 1.91–6.02) for the D/AO group and 1.69 (95% CI: 1.11–2.57) for the D/NAO group (Table 3).

**Table 3:** Worsening disability risk according to dynapenic abdominal obesity and sarcopenic obesity groups, using non-dynapenic non-abdominal obese and non-sarcopenic non-obese, respectively, as reference group.

	**Model 1 (unadjusted)**		**Model 2 (adjusted for age and gender)**		**Model 3 (adjusted for age, gender and other variables)**	
	**Hazard ratio**	**CI (95%)**	**Hazard ratio**	**CI (95%)**	**Hazard ratio**	**CI (95%)**
Dynapenic/non-abdominal obese	1.76	1.20–2.57	1.84	1.25–2.72	1.69	1.11–2.57
Non dynapenic/abdominal obese	2.02	1.39–2.93	1.96	1.35–2.86	2.00	1.34–2.98
Dynapenic/abdominal obese	3.54	2.08–6.01	3.61	2.12–6.15	3.39	1.91–6.02
Sarcopenic/non-obese	0.92	0.70–1.21	0.92	0.71–1.21	0.97	0.73–1.28
Non-sarcopenic/obese	1.13	0.97–1.32	1.12	0.96–1.31	1.10	0.94–1.29
Sarcopenic/obese	1.30	0.65–2.61	1.35	0.67–2.70	1.40	0.69–2.85

Dividing the study population according to relative ASMM/FM% tertiles, no groups showed increased risk of worsening disability (Table 3).

During an average of 5.5 years of follow-up, 133 (48.5%) participants had at least one hospitalization. Mean hospitalizations were 0.6 ± 0.9 and average days in the hospital were 11.9 ± 18.3 days.

Classifying the study population based on relative WC and muscle strength in a Cox regression, and considering all groups with ND/NAO as the reference category, hospitalization risk, after correction for gender and age, was 1.92 (95% CI, 1.12–3.30) for the D/AO group, decreasing slightly to 1.84 (95% CI, 1.06–3.19) after further adjustment for confounding variables (Table 4). The interaction between dynapenia and abdominal obesity in the model was not statistically significant (HR 1.245, 95% CI: 0.773–2.007, *p* = 0.367).

**Table 4:** Hospitalization risk according to dynapenic abdominal obesity and sarcopenic obesity groups, using non-dynapenic non-abdominal obese and non-sarcopenic non-obese, respectively, as reference group.

	**Model 1 (unadjusted)**		**Model 2 (adjusted for age and gender)**		**Model 3 (adjusted for age, gender, and other variables)**	
	**Hazard ratio**	**CI (95%)**	**Hazard ratio**	**CI (95%)**	**Hazard ratio**	**CI (95%)**
Dynapenic/non-abdominal obese	1.09	0.85–1.39	1.08	0.84–1.38	1.11	0.86–1.43
Non-dynapenic/abdominal obese	1.12	0.95–1.31	1.11	0.95–1.31	1.10	0.93–1.29
Dynapenic/abdominal obese	1.96	1.14–3.34	1.92	1.12–3.30	1.84	1.06–3.19
Sarcopenic/non-obese	0.92	0.72–1.17	0.91	0.72–1.16	0.94	0.73–1.20
Non-sarcopenic/obese	1.00	0.86–1.16	0.99	0.85–1.16	0.98	0.84–1.15
Sarcopenic/obese	1.71	0.95–3.12	1.74	0.95–3.16	1.81	0.98–3.33

According to ASMM/FM% tertiles, borderline significance only for S/O was 1.81 (95% CI, 0.98–3.33) (Table 4).

## Discussion

The results of this study show that the prevalence of DAO and SO was 12.0 and 8.0%, respectively, and that the two conditions should be considered as two distinct phenotypes. Interestingly, only two participants, corresponding to 0.7% of the population, were classified as both DAO and SO. This result is in line with those of Scott et al. (22) and their population numbering 674 participants of similar age that found a higher prevalence of dynapenic abdominal obesity (*n* = 79) as compared to sarcopenic obesity (*n* = 57); none of the subjects were both sarcopenic obese and dynapenic abdominal obese.

Moreover, elderly subjects with low muscle strength and abdominal obesity (the so-called dynapenic abdominal obese condition) could be considered at particularly high risk for worsening disability and early hospital admission over a 5.5-year follow-up period.

Our data also suggest that subjects with SO, defined as based on DXA relative to ASMM and FM% measurements, may not present an increased risk of worsening disability.

This study expands on the results previously obtained in the same population (13) and considers hospitalization risk as additional outcome and classifies the study sample on the basis not only of muscle strength and WC, but also of DXA-derived ASMM and FM%.

Our results show no relation between SO and worsening disability in the elderly, in contrast with those observed in the population of the New Mexico Aging Process Study by Baumgartner et al. (8) who demonstrated that community-dwelling elderly with sarcopenic obesity show a more than 2-fold higher risk of IADL disability onset as compared with those who were not sarcopenic obese at baseline evaluation. Some differences between our study and that of Baumgartner et al. (8) should be acknowledged.

Firstly, in the Baumgartner et al. study (8), disability was only evaluated with the IADL scale, while in our study a composite disability score was used, which took into account both ADL and IADL reported difficulties. Secondly, the studies were different in the sample size and length of follow-up.

Our results seem in line with the results from National Health And Nutrition Examination Survey III (23) and data obtained from a sample of women in the Health Aging Body Composition Verona study (24), in which no association between SO and disability was observed. It should be noted that these studies used different methods to define SO (i.e., the cross tabulation of ASMM and FM as evaluated by DXA).

Even though several studies showed association between disability and WC (9–14), alone and combined with muscle strength, no study has so far prospectively compared the prognostic implication of both DAO and SO on disability in older adults.

A strong relation between worsening disability risk and DAO has been shown in previous epidemiological studies (13, 14), without applying the SO classification in the same population.

The main attempt of the present paper is to investigate if the SO phenotype is exposed to a greater risk of worsening disability and hospitalization.

In the present study we also observed that participants diagnosed with DAO, but not with SO, showed an independent negative on hospitalization risk and a shorter time to first hospitalization compared with other groups, even after adjustment for confounding factors.

Our results agree with those of Cawthon et al. (25) from the Health ABC study, who observed that lower extremity extension strength, but not with muscle mass, was associated with the highest risk of hospitalization. This result may be partially explained by the unfavorable metabolic profile, lower vitamin D levels and higher level of inflammation and disability score, showed by DAO subjects at baseline, as compared with other groups. This seems to be partly in line with previous cross-sectional observations from the NHANES and Korean Sarcopenic Obesity Study (26–28), which confirmed the existence of a link between simultaneous presence of abdominal fat deposition, low muscle mass and high sensitivity C reactive protein (hsCRP), low vitamin D levels and insulin resistance, with impact on physical functioning.

We are not aware of any previous studies that have examined the relationship between SO and hospitalization risk, but in our study population an increased risk was observed only in DAO subjects. While in our study definition, SO demonstrated a relationship with hospitalization risk that approached significance, our results suggest that assessment of dynapenic abdominal obesity alone may be adequate for identifying older adults at risk of hospitalization. However, it cannot be ignored that the interaction between dynapenia and abdominal obesity was not significant.

This result expands on the results from the InChianti Study population where the classification of the study population based on DXA variables was not possible due to the lack of DXA measurements (14).

In our study population, the use of muscle strength and fat distribution measurements allowed accurate identification of population at higher risk of adverse events, as compared to SO identification. Moreover, the assessment of ASMM and FM% with DXA require imaging procedures and X-ray exposure that are not portable and easily available, whereas measure of fat distribution with WC and muscle strength with inexpensive handheld dynamometers can be quickly implemented in different clinical settings.

We recognize that there are some study limitations that warrant mention. Firstly, our study population was limited to community dwelling elderly subjects, generally in good health condition at baseline, and thus not representative of a normal aging population.

Secondly, disability was evaluated with a questionnaire and hospitalization record was obtained through their GPs' records and not verified by hospital records.

Thirdly, it is possible that some study groups were too small due to the limited sample size. In particular, the SO subgroup reduced size could have influenced the statistical power of the association with the risk of hospitalization. Therefore, our results must be considered with caution, and our findings need to be confirmed in wider populations with available data for both DXA and waist circumference/muscle strength classifications.

The findings from our study seem to encourage the use of two simple, broadly available measurements such as muscle strength and waist circumference, in order to easily stratify the elderly population for unfavorable health outcomes, including those in outpatient settings.

In conclusion, our results seem to indicate that dynapenic abdominal obese subjects are at higher risk of worsening disability and hospitalization, when compared with non-dynapenic non-abdominal obese. On the contrary, sarcopenic obese subjects, in our study population, did not show a worse risk profile when compared with the non-sarcopenic non-obese group.

Identification of elderly subjects with low muscle strength and simultaneous central fat distribution could help to identify groups of individuals at particularly high risk for unfavorable health outcomes. Interventions designed to improve physical performance and muscle strength and to reduce the prevalence of obesity in the elderly, may not only help in delaying the disability onset, but also be effective in controlling the dramatic economic burden associated with hospitalization.

## Data Availability Statement

The datasets for this manuscript are not publicly available because the disclosure of data was not included in the informed consent signed by the participants. Requests to access the datasets should be directed to Andrea P Rossi, andrea.rossi3@aovr.veneto.it.

## Ethics Statement

The studies involving human participants were reviewed and approved by Azienda Ospedaliera Universitaria Integrata di Verona. The patients/participants provided their written informed consent to participate in this study.

## Author Contributions

AR, FF, and MZ: study concept and design, analysis and interpretation of data, and preparation of manuscript. NN, PB, AB, SU, AM, and VM: interpretation of data and preparation of manuscript. AR, FF, EZ, GM, and MZ: acquisition of subjects, collection of data, and manuscript revision. The manuscript has been extensively revised by Prof. Mark Newman, a native English speaker. All authors contributed to the article and approved the submitted version.

## Conflict of Interest

The authors declare that the research was conducted in the absence of any commercial or financial relationships that could be construed as a potential conflict of interest.
